# An investigation of social media labeling decisions preceding the 2020 U.S. election

**DOI:** 10.1371/journal.pone.0289683

**Published:** 2023-11-15

**Authors:** Samantha Bradshaw, Shelby Grossman, Miles McCain

**Affiliations:** 1 School of International Service, American University, Washington, D.C., United States of America; 2 Stanford Internet Observatory, Stanford University, Stanford, California, United States of America; CCET: Chandigarh College of Engineering and Technology, INDIA

## Abstract

Since it is difficult to determine whether social media content moderators have assessed particular content, it is hard to evaluate the consistency of their decisions within platforms. We study a dataset of 1,035 posts on Facebook and Twitter to investigate this question. The posts in our sample made 78 misleading claims related to the U.S. 2020 presidential election. These posts were identified by the Election Integrity Partnership, a coalition of civil society groups, and sent to the relevant platforms, where employees confirmed receipt. The platforms labeled some (but not all) of these posts as misleading. For 69% of the misleading claims, Facebook consistently labeled each post that included one of those claims—either always or never adding a label. It inconsistently labeled the remaining 31% of misleading claims. The findings for Twitter are nearly identical: 70% of the claims were labeled consistently, and 30% inconsistently. We investigated these inconsistencies and found that based on publicly available information, most of the platforms’ decisions were arbitrary. However, in about a third of the cases we found plausible reasons that could explain the inconsistent labeling, although these reasons may not be aligned with the platforms’ stated policies. Our strongest finding is that Twitter was more likely to label posts from verified users, and less likely to label identical content from non-verified users. This study demonstrates how academic–industry collaborations can provide insights into typically opaque content moderation practices.

## Introduction

On January 6, 2021, after a mob stormed the U.S. Capitol, Facebook announced that it was blocking then U.S. President Donald Trump’s Facebook and Instagram accounts, which prevented him from posting [1]. A few days later Twitter permanently suspended his account [2] and YouTube banned him from uploading any new videos to his channel [3]. Given Trump’s long history of spreading violative content on the platforms, these sudden decisions perpetuated the perception that social media platforms enforce their policies in an ad hoc, inconsistent, and reactionary manner [4]. Media reporting has also suggested that platforms are similarly inconsistent in enforcing policies related to hate speech [5] and misinformation [6, 7].

Methodologically, it is hard to assess platform (in)consistency at scale for two reasons. First, we do not know what content is reported to or flagged by the platforms, which complicates efforts to evaluate their enforcement decisions relative to content that is not escalated for review. Second, platforms’ terms of service can impede certain types of data collection, such as scraping [8].

We leverage a novel cross-platform dataset of 1,035 social media posts pushing 78 misleading election-related claims posted during the 2020 U.S. presidential election that were reported to Facebook or Twitter to quantify how and when platforms labeled content. Our dataset comes from the Election Integrity Partnership, a coalition of four civil society groups (the Stanford Internet Observatory, the University of Washington’s Center for an Informed Public, Graphika, and the Atlantic Council’s Digital Forensic Research Lab), that collaborated with social media platforms ahead of the 2020 election. (See the S1 Appendix for details on our involvement with the Partnership.) The Partnership flagged misleading online content that could undermine the election and appeared to violate social media platform policy, and shared it with platforms that had staff monitoring Partnership reports. Drawing on this unique data set, we assess the extent to which platforms consistently take action against misleading narratives. Due to the difficulty of assessing whether a platform or user removed content, we focus on whether live content was *labeled*.

We find that for 69% of the misleading claims, Facebook handled posts sharing the claim consistently. For the remaining 31% of claims, Facebook treated posts on its own platform sharing the same claim inconsistently. The findings for Twitter are nearly identical: 70% of claims were labeled consistently, and 30% inconsistently. We investigated these inconsistencies, and found that most were arbitrary based on information about the posts that was visible to the public. In roughly one-third of the cases (28%) we found plausible explanations for the inconsistent labeling, although these reasons may not be aligned with stated policies. For example, Twitter was more likely to label posts from verified users, and less likely to label identical content from non-verified users.

Social media platforms control which misleading election content is moderated and which is not. These enforcement decisions are critical to election integrity, but we lack a descriptive understanding of enforcement consistency. This paper investigates content moderation practices to identify patterns. We also demonstrate how academic–industry collaborations can provide insight into these typically opaque practices.

In what follows we discuss how prior work has measured content moderation in practice, and what we know about the effect of labeling content. We then provide an overview of Facebook’s and Twitter’s relevant content guidelines ahead of the 2020 election. Next we discuss the social media post dataset, and how we measure labeling. We then look at labeling variance, according to user type and post attributes.

## Measuring enforcement and its impact

Content moderation is one of the most important areas of platform governance: moderation decisions have implications for the freedom of speech, information access, and online participation [9–14]. Yet such decisions often occur in a black box. Obscured by algorithms and an invisible workforce of human moderators, day-to-day decisions about content are often concealed from public scrutiny and oversight [13, 15].

What we do know about platform content moderation policies and enforcement comes from a variety of sources, including platform community guidelines, interviews with content moderators [16] or current and former platform employees [17], public disclosures from platforms on coordinated disinformation [18, 19], company transparency reports [20], reporting on specific types of problematic content such as child sexual abuse material [21], and, more recently, rulings from the Facebook Oversight Board [22]. These diverse sources have revealed that content moderators must often make decisions quickly, sometimes without sufficient context, and at other times without enough capacity to address the volume of harmful content surfacing in specific communities or regions [23, 24]. For certain platforms and particular kinds of content, we have also gained insights into the amount of violative content removed. And repeated disclosures of inauthentic disinformation campaigns from Facebook and Twitter have enhanced our understanding of how platforms define such operations.

Several studies have examined how enforcement mechanisms affect the spread of misinformation and disinformation online. In this paper we focus on “soft moderation” which involves attaching a warning or information label to content that could be harmful or misleading [25, 26]. This is distinct from “hard moderation,” which involves removing a post or suspending an account. Previous experimental work has demonstrated that placing warning labels on social media posts reduces individuals’ willingness to share information [27] and makes them less likely to believe an article is accurate [28]. There are risks, however, if true information is incorrectly labeled as false, or if false claims go unlabeled [29, 30]; exposure to labels in general may decrease trust in *true* news stories [28]. While some analysts have theorized about a supposed “backfire effect” in which corrective information about inaccurate information that individuals are inclined to believe instead causes people to dig in their heels and believe the story more strongly, there is evidence that this risk has been overstated [31]. While these studies provide insights into the *effects* of these moderation decisions, they do not generate a complete picture of *when* platforms label or remove content.

To evaluate enforcement, we would need to have a denominator—a sense of the universe of misleading election-related content—in addition to the amount of content actioned. Transparency reports provide some insights into the latter, but do not offer the granularity needed to scrutinize enforcement decisions [32]. The dataset we introduce in the data and methods section allows us to partially circumvent these methodological issues. Because we know that platform representatives reviewed all social media posts in the dataset, we can be confident that all content in the dataset—whether it was labeled or not—was seen.

## Social media, enforcement, and the 2020 U.S. election

Ahead of the 2020 election, social media platforms changed and clarified their policies related to what election-related content was (and was not) acceptable. The Election Integrity Partnership focused on identifying, flagging, and analyzing misleading content related to calls to violent action, delegitimization of the results, participation interference, procedural interference, and fraud. These categories are defined in the S1 Appendix. The S1 Appendix, which draws on the Election Integrity Partnership report [33], shows that Facebook and Twitter at a minimum *mentioned* prohibitive policies about each of these categories. However, reasonable people could disagree about whether platforms *should* have actioned all content the Partnership flagged, and we do not take the position that platforms should have actioned all content in our dataset.

We avoid using the term “misinformation” to describe social media posts in the dataset, instead referring to them as “misleading.” While misinformation is sometimes defined as misleading or deceptive information [34], the public often understands the term to mean falsifiable and false information. While many of the posts we analyze are false, others shared claims that were so broad as to be unfalsifiable.

We investigate three research questions. If the perceived arbitrary nature of platform enforcement stems from inconsistency in what content is escalated for review, one might expect consistent within-narrative enforcement given that our sample includes only content that we know has been reviewed. Alternatively, inconsistency could be the result of unclear internal content moderation policies, which would still be a factor in the posts in our dataset.

**Q1**: To what extent will platforms label consistently within the same narrative?

We investigate whether enforcement varies based on whether the user is verified. Posts from verified users may be less likely to be labeled given the newsworthiness exemptions granted to politicians on both platforms prior to the 2020 election [35, 36]. While these exemptions explicitly applied to decisions about whether to remove content, they could also be relevant to labeling decisions. Alternatively, posts from verified users may be subject to additional scrutiny due to their large audience, making their posts more likely to be flagged, which could make their posts more likely to be labeled than similar posts from non-verified users [37].

**Q2**: Will posts from verified users be more or less likely to be labeled compared to similar posts from non-verified users?

On the one hand, post labeling may not vary by language, as platform policies around the U.S. 2020 election theoretically applied equally to content in all languages. But on the other hand, posts in English may be more likely to be labeled if there are more qualified content moderators to review such content, and some research shows examples of English tweets being labeled and similar tweets in other languages not being labeled [26]. Posts sharing external URLs, images, or videos may be more likely to be labeled if it is easier for platforms to automatically flag such content, or such content may be less likely to be labeled as text might be easier to review.

**Q3**: Will labeling vary depending on post-level factors, including whether it is in English or shares a URL, image, or video?

## Data and methods

Our dataset comes from the Election Integrity Partnership, a coalition of civil society groups that “was formed to enable real-time information exchange between election officials, government agencies, civil society organizations, social media platforms, the media, and the research community. It aimed to identify and analyze online mis- and disinformation, and to communicate important findings across stakeholders” [38]. The Partnership was operational from July 2020 to February 2021 [33].

The Partnership used Jira, a work tracking and management platform, to create tickets highlighting misleading narratives about the November 2020 election. Relevant content included misinformation about election procedures, content potentially deterring participation in the election, content encouraging electoral fraud, content trying to delegitimize the election results based on misleading claims, and calls to violent action. A team of 97 people worked in shifts from September 3 to November 19, 2020 to identify this content on Discord, Facebook/Instagram, Google/YouTube, NextDoor, Periscope, Pinterest, Reddit, TikTok, and Twitter. Analysts identified misleading content by manually monitoring lists they had created of public groups or accounts. For example, one analyst focused on content in military/veterans communities. They created a list of accounts across platforms to monitor by searching for a set of 12 keywords. During their shift, they manually reviewed posts on these accounts. The final ticket-level (or narrative-level) dataset included 557 tickets in scope for this research, which is a smaller number than the overall number of incidents that the Partnership studied.

**Fig 1:**
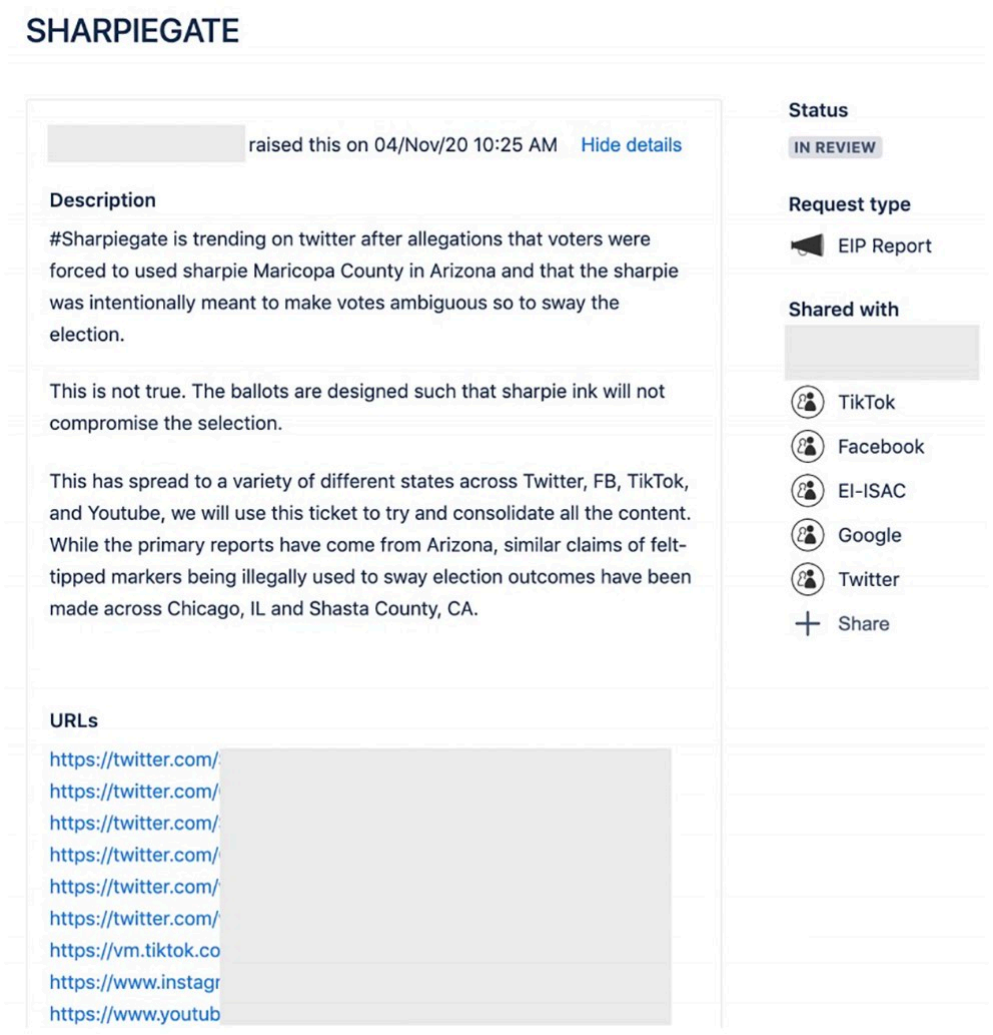
Example ticket on the Jira platform, from the Partnership final report [33]. The greyed-out URLs are example posts. The platforms listed under “Shared with” were alerted about the ticket. In a comment section on the ticket (not shown) platform partners would respond with, e.g., “Received,” “Thank you” or “Reviewing this content on our side.”

Each ticket included at least one social media post linking to instances of misleading content. Once the ticket was filed, a Partnership manager would tag partner platforms to alert them to the misleading content. While we do not make the normative claim that all the posts in the tickets necessarily “warranted” removal or a label, all were deemed sufficiently misleading and potentially violative of platform policy that the Election Integrity Partnership referred them to the platforms for review on a case-by-case basis. Partner platforms on the Jira ticketing system included Facebook/Instagram, TikTok, Twitter, and Google/YouTube. Platforms would confirm receipt of the alert, and typically indicate that they were investigating the ticket. See Fig 1 for an example ticket.

We first subset our post-level dataset to live posts because we were frequently unable to determine whether unavailable posts had been removed by the user or the platform. We then identified misleading claims that had at least five live posts on a given platform. Ultimately, only Facebook and Twitter had a sufficient number of narratives that met these criteria to merit analysis (n = 111). We removed 33 of these narratives on the grounds that they were vague enough to potentially cover more than one narrative. For example, one ticket was dedicated to rejected ballots, but included posts covering different claims across states and voting methods about rejected ballots.

We extracted all social media posts from the remaining 78 claims, and in June 2021 manually inspected each post. We removed a small number of posts that should not have been included on a given narrative, as well as non-posts (e.g., groups or profiles), which generated a final analysis dataset of 1,035 posts (603 on Twitter and 432 on Facebook). In short, at least two individuals reviewed each post to assess whether it was categorized under the correct narrative: first, a Partnership member who assigned the post to a narrative in real time, and second, an author of this paper who reviewed the decision a few months later. In cases of disagreement between the real time and delayed coding, all three authors discussed the post and came to a consensus.

Our process for coding whether a post was labeled also involved two levels of coding. We first ran an automated Python script that took an archival screenshot of each post and detected whether or not that content had been labeled. At least one author of this paper then manually reviewed each archival screenshot to verify that the script’s classification was correct. The script performed reasonably well, making mistakes just 3.5% of the time, mostly due to multiple tweets appearing on the same page (e.g., in a thread). For more information about this automated script see the S1 Appendix. The S1 Appendix also contains a selection of labels that both platforms applied to election-related content.

We also coded whether the user was verified, the post language, and whether the post shared a URL, image, or video. Both platforms used a variety of labels, ranging from “Stay informed: Learn about US 2020 election security efforts [link]” to “Missing context: The same information was checked in another post by independent fact-checkers.” We do not distinguish between label types in our analysis because 1) the number of different labels used would limit our statistical power and 2) all labels appear to have been used punitively, applied only to misleading posts. We note that our data collection and analysis methods complied with the terms and conditions for the various data sources.

Our dataset has at least three limitations. First, we focus on Facebook and Twitter because we lacked sufficient posts on other platforms to conduct variance analysis. The Election Integrity Partnership dataset contained a plurality of Facebook and Twitter posts due to the existence of tooling that made it easier to find and analyze content on these platforms. Many important platforms are not included in our dataset due to unevenness in data access or because the platform did not partner with the Partnership *on the Jira ticketing system*. Second, the posts in our dataset are not exhaustive. Given the scale of election-related content, the Partnership did not attempt to track every instance of each narrative online. The dataset instead includes examples of the narratives. Third, we focus only on the decision to label live content. Therefore we cannot draw conclusions about the prevalence of overall content actioning, as we excluded content that a user deleted, or that a platform suspended.

## Results

### Do platforms label consistently within a narrative?

**Fig 2:**
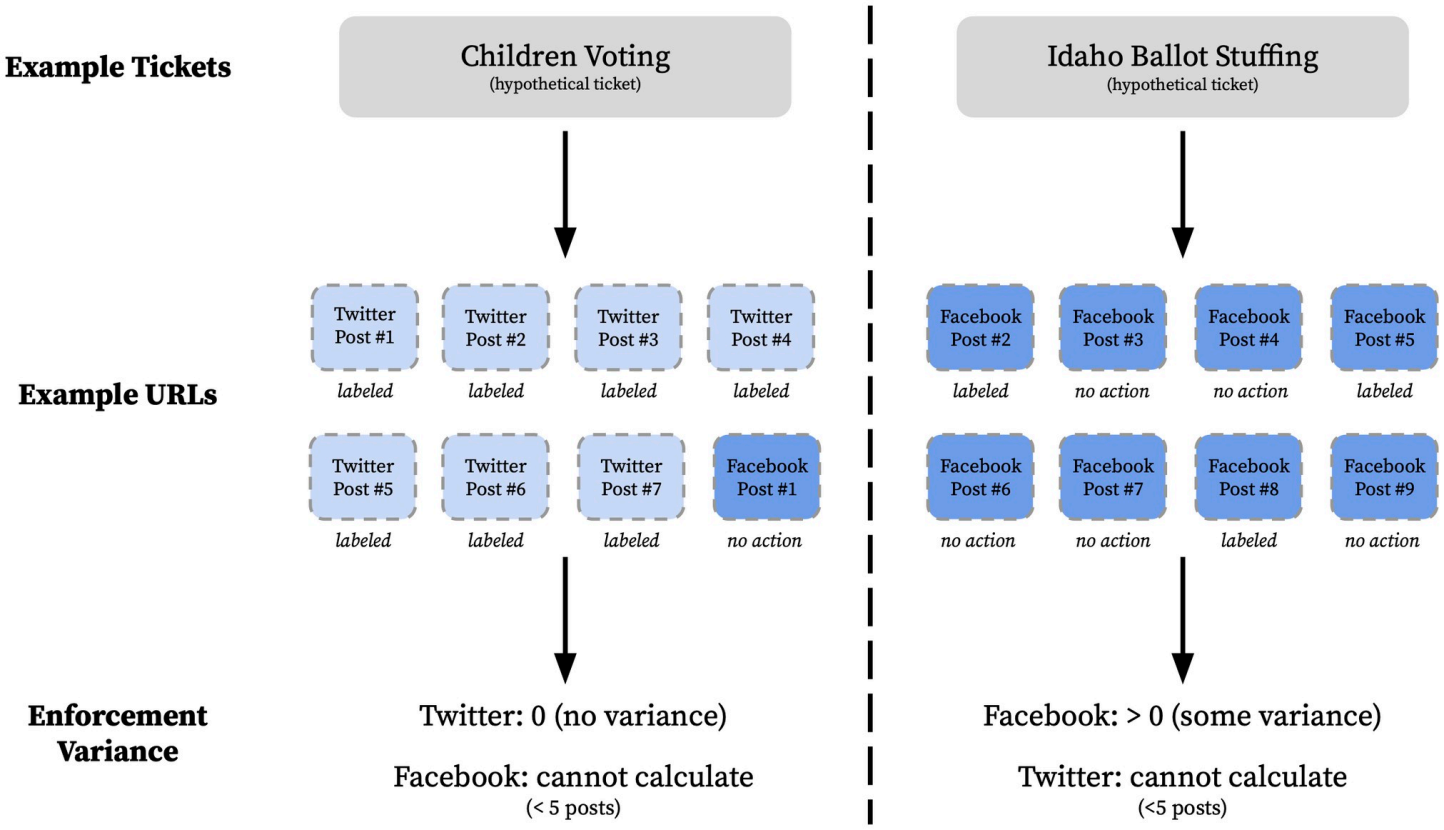
Ticket schematic. A stylized overview of the dataset and variance calculation.

Fig 2 displays a stylized overview of the dataset structure and variance calculation.

We note that this is not a perfect test of platform consistency *in general*, as two tweets, for example, could spread the same misleading content, but one could be far more harmful than the other. Likewise, platforms’ enforcement policies could be based on tactics or account-level information (which could be public or private), which could vary within a claim.

Table 1 reports our first finding. For 69% of the misleading claims on Facebook, and 70% of those on Twitter, the platforms consistently labeled each post that included one of those claims, always either adding a label or not. For the remaining 31% of misleading claims on Facebook, and 30% on Twitter, both platforms inconsistently labeled each post that included the same claim.

**Table 1:** Portion of narratives with and without any variance in labeling by platform. Approximately one-third of narratives had at least some labeling variance on both Facebook and Twitter.

	Facebook	Twitter
No labeling variance	0.69	0.70
Some labeling variance	0.31	0.30

### What are the correlates of platform enforcement?

**Fig 3:**
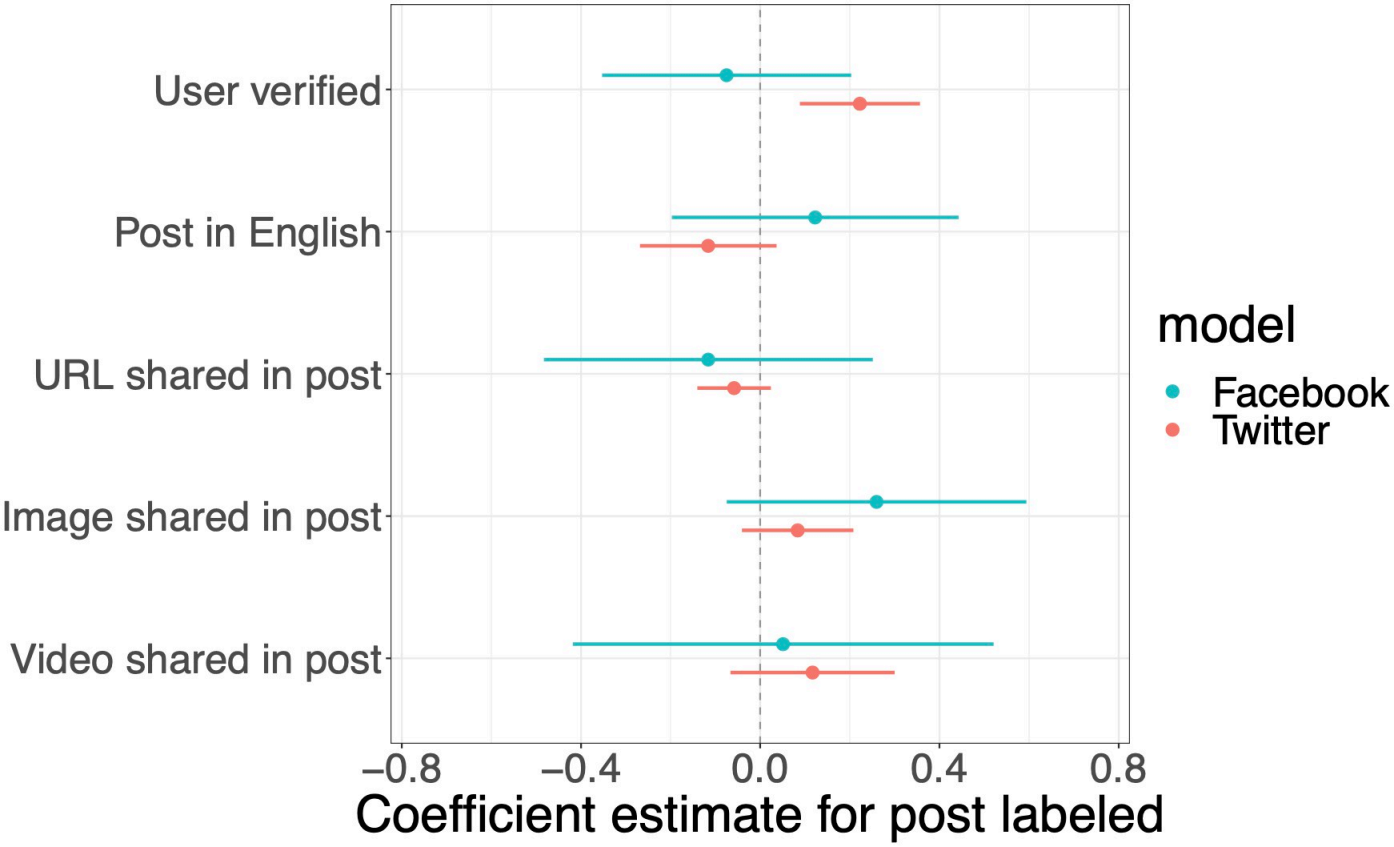
Relationship between post-level attributes and whether the platform labeled the post. Standard errors are clustered at the claim level.

Next we evaluated the relationship between user and post attributes and whether the post was labeled. We used ordinary least squares models (one model for Facebook and one for Twitter) and clustered standard errors at the narrative level. We look at the relationship between several binary explanatory variables: whether the user was verified, whether the post was in English (the S1 Appendix shows the number of posts by language by platform), and whether they were labeled; 93% of the posts were in English, and the remainder were in 11 different languages), whether the post included a URL, whether the post included an image, and whether the post included a video. The outcome variable, also binary, was whether the platform labeled the post. Clustering standard errors at the narrative level (i.e. the level of the misleading claim) allowed us to maximize the statistical power of the URL-level dataset. Fig 3 shows that Facebook was no more or less likely to label posts from verified users, and that Twitter was 22% more likely to label tweets from verified users. We found no relationship between the other explanatory variables and labeling for either platform.

### Is there a pattern to labeling variance within the same narrative?

Are the narratives that Facebook and Twitter appear to have treated inconsistently actually being treated inconsistently? Or are there patterns in which posts that make the same claim are and are not labeled? We investigated all platform–claim dyads with any labeling variance to better understand whether the variance is explainable. Table 2 displays our findings.

**Table 2:** Summary of narratives with labeling variance.

Platform	Narrative	Pattern or arbitrary	Pattern explanation	Number posts	Number posts labeled	Portion posts labeled
Facebook	Some counties have more registered voters than people eligible to vote.	Arbitrary		7	1	14.29%
Facebook	A GOP poll watcher was denied entry to a polling place.	Arbitrary		6	5	83.33%
Twitter	GOP election observers intimidated voters in Detroit.	Arbitrary		12	1	8.33%
Twitter	Project Veritas whistleblower from a USPS office alleged a postmaster forced backdating of ballots.	Arbitrary		136	1	0.74%
Facebook	Congressman Clay Higgins claimed Donald Trump won the election.	Arbitrary		5	2	40.00%
Facebook	A whistleblower at the Clark County Elections Department in Nevada claimed poll workers processed illegitimate ballots.	Arbitrary		5	2	40.00%
Twitter	Benford’s law is evidence of voter fraud.	Arbitrary		26	4	15.38%
Facebook	A call for Antifa members to dress up as Trump supporters or police so that if Antifa needed to use violence it wouldn’t lose popular support.	Arbitrary		5	4	80.00%
Facebook	Biden’s Texas political director participated in a ballot harvesting scheme in Harris County, Texas.	Arbitrary		8	2	25.00%
Twitter	Ilhan Omar-linked individual paid someone $200 to vote.	Arbitrary		7	1	14.29%
Twitter	Twitter supressed Donald Trump’s tweet, so other users should reshare it verbatim.	Arbitrary		11	1	9.09%
Facebook	138,000 Biden votes and no Trump votes were found in Michigan, indicating widespread fraud.	Arbitrary		5	3	60.00%
Twitter	138,000 Biden votes and no Trump votes were found in Michigan, indicating widespread fraud.	Arbitrary		10	4	40.00%
Facebook	There were more votes than registered voters in Wisconsin.	Arbitrary		55	54	98.18%
Twitter	Nevada GOP claimed ballot fraud could easily occur.	Arbitrary		5	4	80.00%
Facebook	Trump declared the victor in Pennsylvania.	Arbitrary		6	1	16.67%
Facebook	Democrats used ballot drop boxes as part of an overarching plan to cause chaos.	Arbitrary		12	10	83.33%
Twitter	A software glitch was evidence of voter fraud.	Arbitrary		7	2	28.57%
Twitter	Voters in Maricopa County were forced to use Sharpies while voting in a ploy to invalidate their ballots.	Pattern	Content mentioning “SharpieGate” explicitly was almost always labeled, as was content from verified accounts. Content not explicitly mentioning SharpieGate and content from non-verified accounts was not labeled.	14	5	35.71%
Twitter	Philadelphia destroyed mail-in ballots to make an audit impossible.	Pattern	Tweets from verified users were labeled, while those from unverified users were not.	5	1	20.00%
Twitter	CIA planned to alter vote results by hacking voting machines.	Pattern	Tweets that used both “Hammer” and “Scorecard” were labelled, but those that did not include both words (such as “Operation Scorecard”) were not labelled.	9	7	77.78%
Twitter	Poll workers in Delaware County filled in empty ballots.	Pattern	Videos that were embedded into tweets were labelled. Videos that appeared as a URL, where a user would have to click on the link to view a video, were not labelled.	5	3	60.00%
Twitter	Senator Thom Tillis claimed to win the election before it was called.	Pattern	Tweets from verified users were labeled, while those from unverified users were not.	5	1	20.00%
Twitter	Queens Village, New York residents received ballots that were pre-filled out for Biden.	Pattern	The labeled tweets showed a video or image of a large portion of the pre-filled ballot, while the unlabeled tweet showed an image of a more generic portion of the ballot, though the text of the tweet pushed the same narrative as the labeled tweets.	6	5	83.33%
Facebook	Benford’s law is evidence of voter fraud.	Patttern	All labeled posts shared the same URL. Other posts sharing similar claims were not labeled.	64	6	9.38%

Of the 11 Facebook claims that had labeling variance, 10 appeared to be arbitrary. Put differently, the labeling variance was unexplainable using publicly available information. Of the 14 Twitter claims with labeling variance, we assessed that eight were arbitrary. For both platforms, a handful of claims included in Table 2 have very low labeling variance; most posts were treated identically.

For the claims that had labeling variance for which we could identify a pattern, we argue that these patterns were never aligned with the platforms’ stated policies. For example, tweets using the hashtag #SharpieGate were labeled, while those similarly claiming that using Sharpies could invalidate your ballot, but without using that hashtag, were not.

### Patterns in labeling variance

In this section we describe three patterns we observed while investigating labeling variance within the same platform, for the same narrative. First, we found claims where Twitter labeled posts from verified users, but not unverified users, which is consistent with the findings in Fig 3. Second, we found narratives in which Twitter labeled tweets with a particular keyword, but not those making the same claim without using the keyword. Third, we identified cases on Twitter in which slight variations in the image or video shared changed whether the tweet was labeled. While these are examples of explainable enforcement variance, they are not necessarily aligned with platforms’ stated policies.

#### Verified user tweets labeled, unverified user tweets not labeled

**Fig 4:**
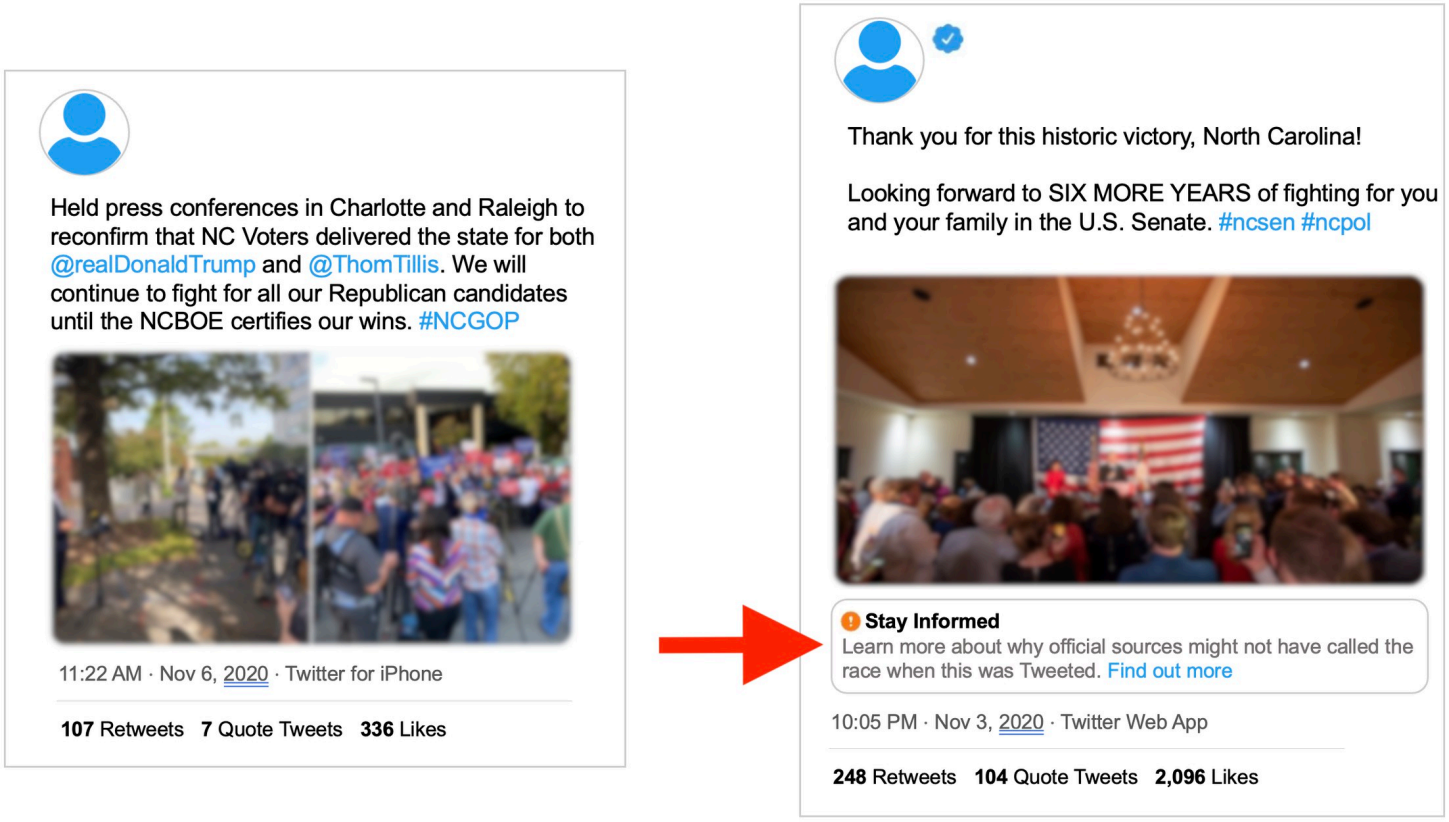
Comparison of verified and unverified content. Left: An unlabeled tweet from an unverified Twitter user. Right: A labeled tweet from a verified Twitter user.

For two narratives on Twitter with labeling variance, tweets from verified users were labeled and those from unverified users were not (see Fig 4). For example, five tweets in our dataset involved claims that Senator Thom Tillis of North Carolina won the election, before the election was called. Only one tweet, from a verified user (Thom Tillis), was labeled. The race was not called until November 11, 2020, several days after these tweets.

#### Reliance on keywords

**Fig 5:**
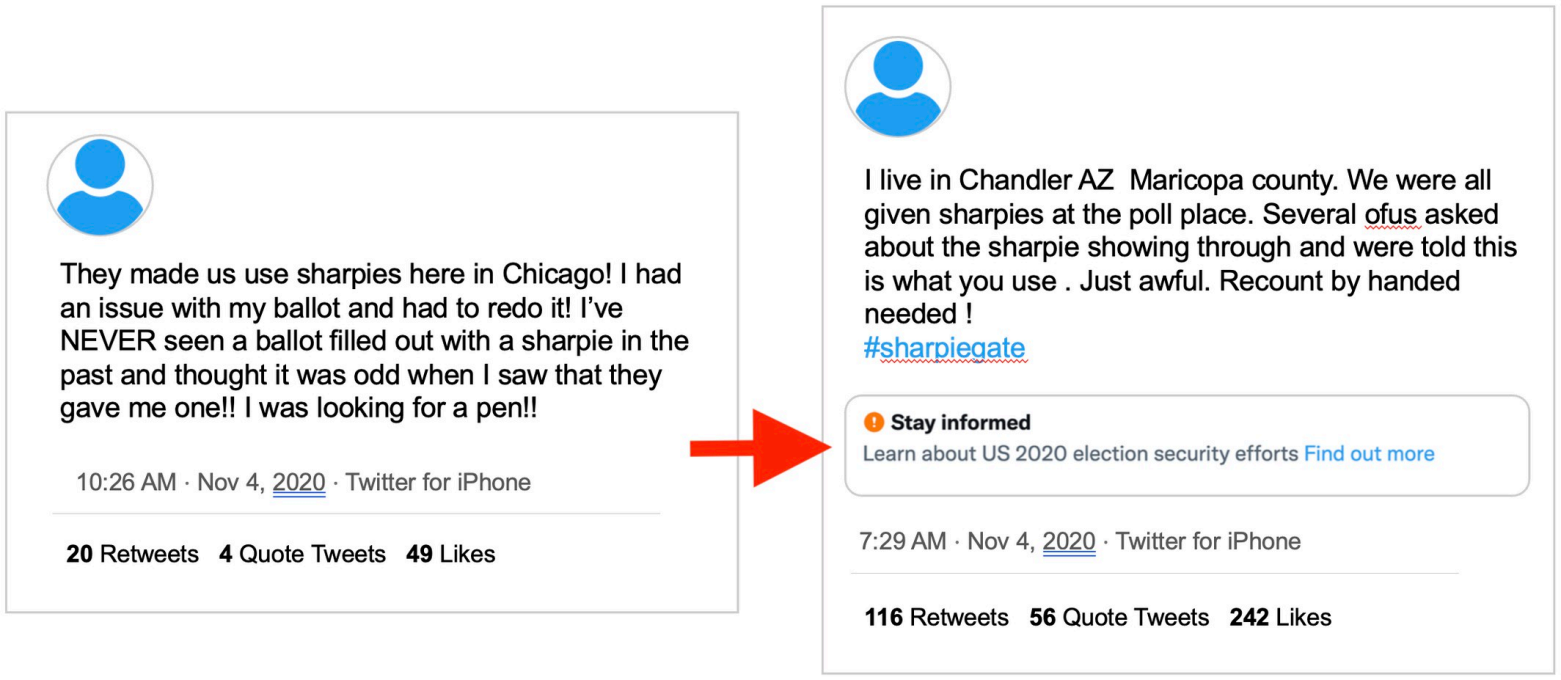
Comparison of keyword and non-keyword tweets. Left: An unlabeled tweet raising questions about the validity of ballots completed with a Sharpie. Right: A labeled tweet raising questions about the validity of ballots completed with a Sharpie that uses the term SharpieGate.

When Twitter treated tweets pushing the same claim inconsistently, we observed that tweets containing a prominent keyword about a narrative were labelled, while tweets spreading the same claim but lacking a specific keyword were not. For example, tweets that used the term “SharpieGate” were almost always labeled, while tweets that falsely claimed voters were using Sharpies to invalidate ballots that did not use the term “SharpieGate” were not labeled (see Fig 5). In another example, tweets that contained the keywords “Hammer” and “Scorecard” were labelled, but those that only included one of these terms—such as tweets using the phrase “Operation Scorecard”—were not labelled. Hammer and Scorecard were the names of two supposed CIA hacking programs.

#### Slight differences in media shape enforcement

**Fig 6:**
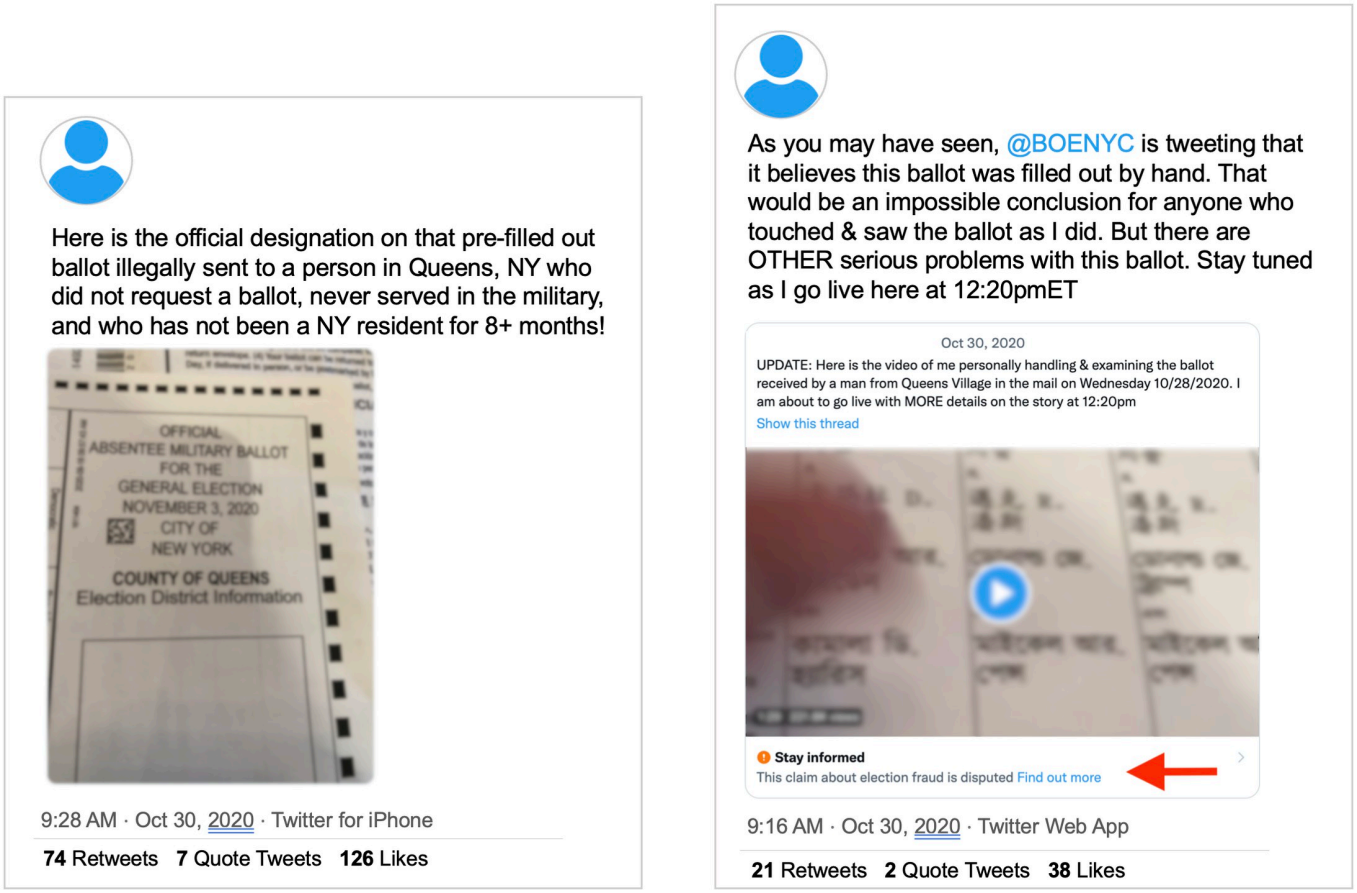
Two differently-labeled tweets with slightly different media. Left: An unlabeled tweet claiming the user saw a pre-completed ballot, and includes an image of part of the ballot. Right: A labeled tweet, making the same claim with a video, that shows a different part of the ballot.

A third pattern we identified on Twitter related to labelling tweets with images and videos. Images were sometimes inconsistently labelled, especially if they were cropped or edited. In one example, an image of a supposedly “pre-filled ballot” received by a Queens, New York resident was shared and labelled by Twitter. However, similar images that cropped the photo, or did not show a complete image of a “pre-filled ballot,” were not labelled, even when they spread the same claim (see Fig 6).

Similarly, Twitter did not always consistently label identical video content. Several tweets shared a video claiming that poll workers in Delaware completed ballots. When the videos were embedded in the tweet such that a user could view the video from Twitter, the tweet was labeled. However, these labels disappeared if the same video was shared as a link to an external website.

### Arbitrary variance in enforcement

While the variance in enforcement against certain narratives was explainable, enforcement against other narratives was not. For example, there were several examples of the exact same content—an image, a video, or an external URL—being treated differently. This may highlight the extent to which Facebook and Twitter struggle to consistently apply their policies.

#### Resharing Trump’s actioned tweet

**Fig 7:**
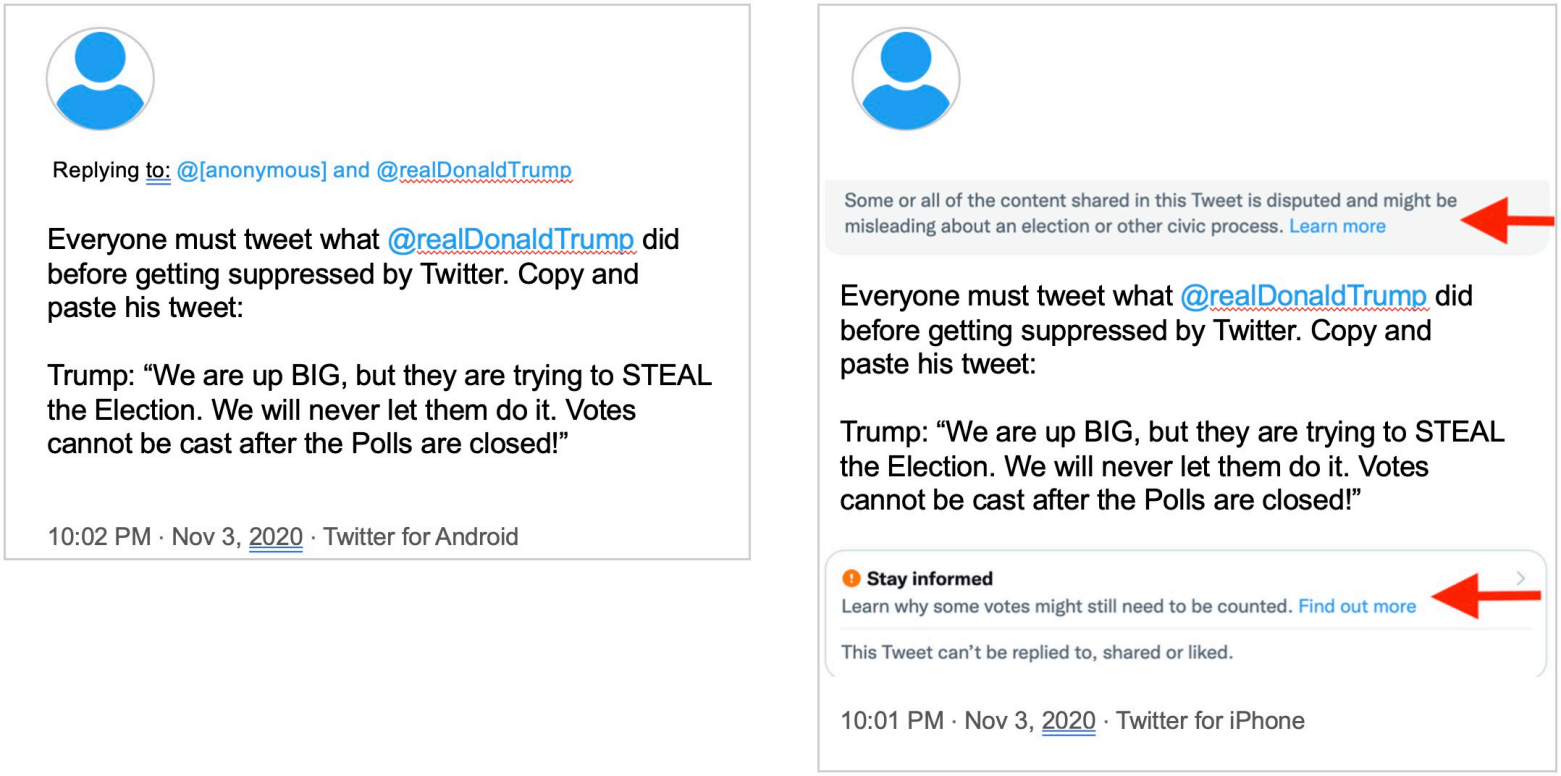
Comparison of inconsistently labeled tweets. Left: An unlabeled tweet resharing Trump’s narrative. Right: A labeled tweet resharing Trump’s narrative.

On November 4, 2020, Donald Trump tweeted that “We are up BIG, but they are trying to STEAL the Election. We will never let them do it. Votes cannot be cast after the Polls are closed” [39]. Twitter quickly actioned the tweet, applying both a “soft-block” warning and a misinformation label [40]. In response to Twitter’s action, a number of Trump’s supporters began reposting his tweet themselves. Twitter applied warning labels to several of these tweets, but did not action others (see Fig 7).

Notably, most of these tweets were posted between 10:00pm and 10:10pm on November 3rd, ruling out the possibility that time explains the differences in enforcement. Moreover, every tweet in this ticket shared Trump’s tweet *verbatim*, making it trivial to detect and surface additional examples for moderation.

#### ‘Finding’ votes in Michigan

**Fig 8:**
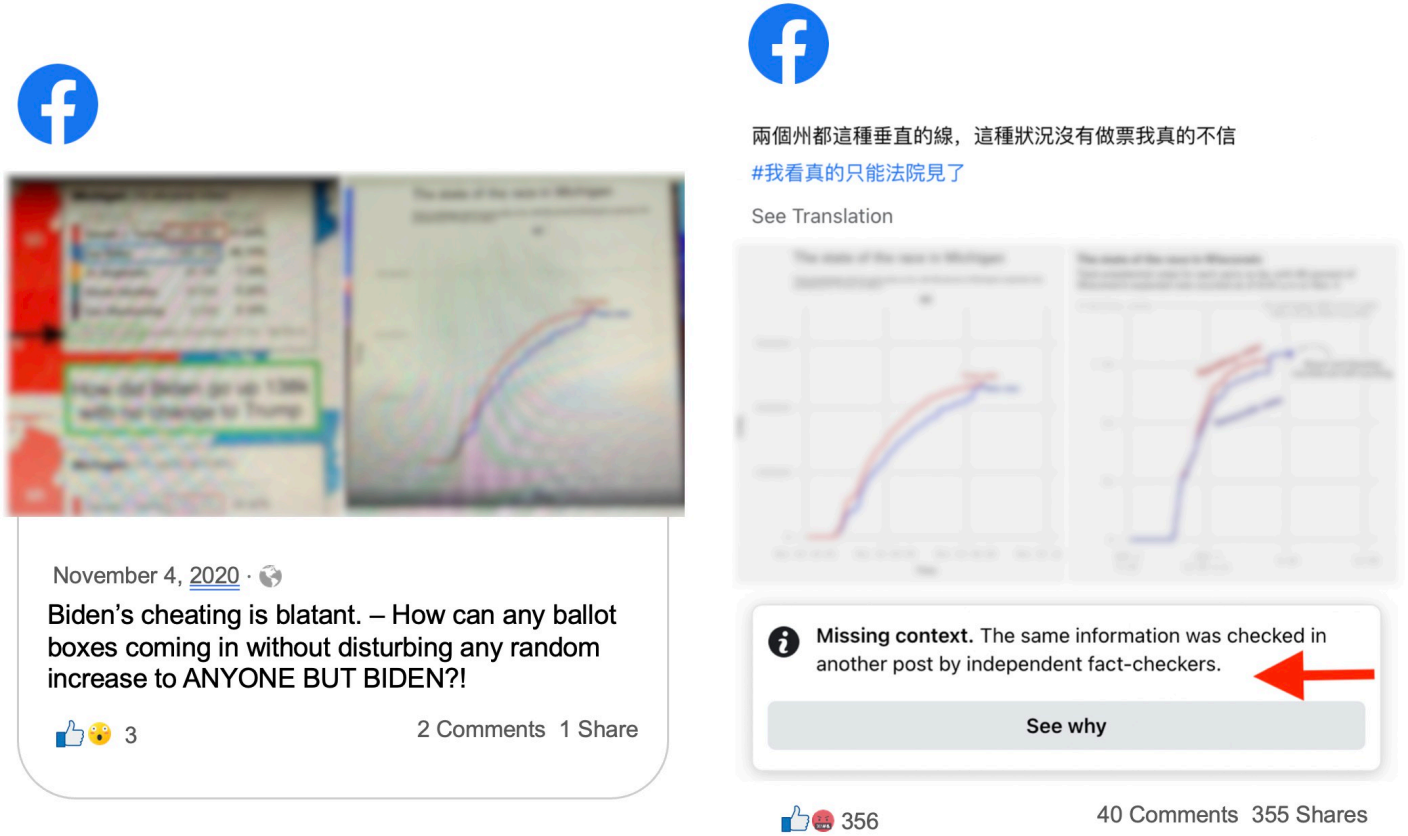
Comparison of differently labeled Facebook posts. Left: An unlabeled Facebook post resharing the misleading narrative. Right: A labeled Facebook post spreading the misleading narrative.

On November 4, 2020, a series of Facebook posts falsely claimed that Democrats “found” 130,000 Biden votes in Michigan. Many of the posts which belonged to this narrative were in Chinese; the S1 Appendix provides more information about our multilingual data and how we analyzed it. While Facebook labeled several of these posts, it did not label others. Language does not explain the difference in enforcement; of the three Chinese-language posts, two were labeled and one was not. Indeed, one of the labeled posts shared the same image as one of the unlabeled posts (see Fig 8).

#### Debunked ‘Antifa’ flyer

**Fig 9:**
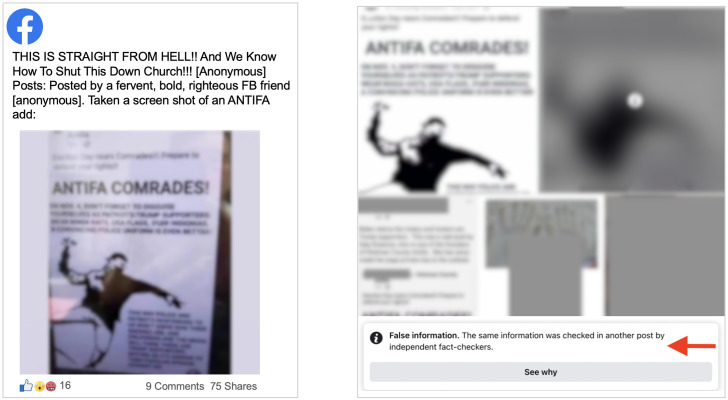
Comparison of differently-labeled Facebook posts about a debunked flyer. Left: An unlabeled Facebook post sharing the debunked flyer. Right: A labeled Facebook post resharing the debunked flyer.

One narrative falsely claimed that a flyer called for “Antifa comrades” to disguise themselves as “patriots/Trump supporters” in an effort to influence public opinion. The flyer has been debunked [41]. This case exemplifies the various ways in which a single piece of media—in this case, a false flyer—are mangled as they spread online. Some of the images are low-resolution screenshots, while others are photos of phones displaying the original flyer. This narrative also contained an extreme example of enforcement inconsistency: One post contained two almost-identical images, only one of which was directly marked as “false information” and blurred. While the non-blurred images are not identical copies of the blurred image—perhaps explaining the inconsistency—they nonetheless feature the same false flyer (see Fig 9).

#### Debunked Benford’s law speculation

**Fig 10:**
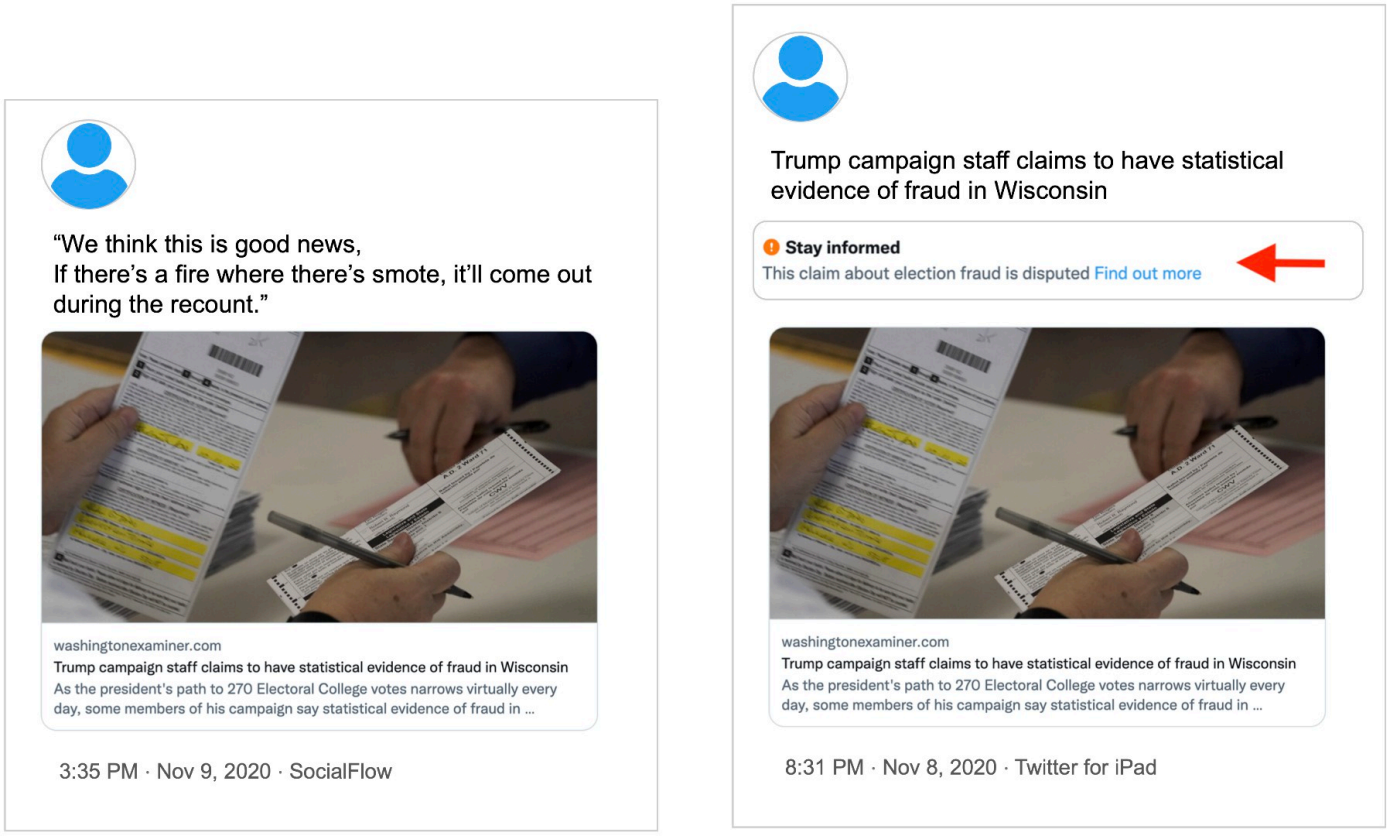
Comparison of differently-labeled Facebook posts about a debunked claim. Left: An unlabeled tweet sharing the debunked claim. Right: A labeled tweet sharing the debunked claim. Note that both tweets linked to the same article.

Finally, one ticket falsely claimed that Benford’s law indicated Democratic voter fraud. Benford’s law is the observation that certain digits are more common in authentic data sets, and it is a real phenomenon. This narrative, however, was misapplied to election data, incorrectly substantiating the idea that Democrats were forging votes. Many of the Twitter posts in this narrative shared a link to the same articles by the Gateway Pundit (a far-right news website that spreads false information [42]) or the Washington Examiner. Interestingly, while Twitter labeled several of the posts *linking* to the Washington Examiner article, it did not apply a label when the Examiner tweeted the article directly (see Fig 10).

Together, these examples illustrate the degree to which Facebook and Twitter likely struggled to consistently apply their policies. Indeed, while much of the conversation around online content moderation focuses on the contours of the policies, platforms’ ability to consistently enforce those policies deserves equal scrutiny.

## Discussion

Our findings suggest that once platforms were made aware of misleading content, they treated narratives consistently more than two-thirds of the time. Yet there were many examples of what appear to be arbitrary enforcement. It is difficult to determine what may have caused this. While we recognize the challenges associated with moderating content at scale, these inconsistencies cannot be attributed to variations in what content was flagged for moderators; in most cases, the platforms were alerted to similar posts at the same time. One possibility is that each post was sent on to individual content moderators separately, who then made different evaluations. If this is the case, it suggests the need for further moderator training, more explicit moderation guidelines, or less time pressure imposed on moderators [16].

Additionally, it appears that Twitter was more likely to label content from public, verified individuals. We are not sure why this is the case. It is possible that user reporting—a variable we lack insight into—was higher for public figures, but we know that Twitter reviewed all tweets in our dataset, which included tweets from both verified and unverified users. It is also possible that Twitter had an internal policy that provided a lower threshold for labeling tweets from verified users due to the greater risk that their content would go viral. We believe we are justified, however, in describing these enforcement differences as “inconsistencies” as we suspect the average social media user believes that if two people post identical content, their posts should receive the same treatment.

We identified many cases in which platforms treated identical or nearly identical content differently. It is not clear to us why platforms would not create a protocol to automatically treat all instances of a news URL or video in the same way.

Last, more research is needed on the effects of the (often fairly generic labels) platforms applied around the U.S. election. For example, one Facebook label occasionally applied to misleading content simply said “See the latest updates on the 2020 US Election.” A Twitter label said “Learn how voting by mail is safe and secure.” Future studies should investigate whether voters perceive such messaging as an implicit critique or perhaps endorsement of the content. Platforms can enable these studies by providing researchers with access to removed and labeled content, for example through their APIs. A broader set of researchers could then investigate content moderation decisions.

## Supporting information

S1 Appendix Appendices: An investigation of social media labeling decisions preceding the 2020 U.S. election. (PDF)
