# Supplementary material for: An investigation of social media labeling decisions preceding the 2020 U.S. election
Source: PLoS One. 2023 Nov 15;18(11):e0289683. doi: 10.1371/journal.pone.0289683 (PMC10650990; doi:10.1371/journal.pone.0289683)
Supplement: S1 Appendix — (PDF) [file pone.0289683.s001.pdf]

Appendices: An Investigation of Social Media Labeling Decisions Preceding the 2020 U.S. Election

August 10, 2023

A. Relationship between authors and Election Integrity Partnership

We, the authors of this paper, all participated in the Election Integrity Partnership in 2020 and 2021 in our capacity as a postdoctoral fellow, research scholar, and research assistant with the Stanford Internet Observatory. It is through our participation with the Partnership that we were able to access a de-anonymized version of the dataset used in this paper. In publishing this paper, we are making public an anonymized version of the dataset and replication code: https://dataverse.harvard.edu/dataset.xhtml?persistentId=doi:10.7910/DVN/YPQ7GP. Due to our API agreement with Meta-owned CrowdTangle, which is signed by Stanford University, we are not permitted to make public the content URLs. We are following political science norms in not making public tweet URLs, as the majority of these tweets are from private individuals. Our anonymized URL-level dataset includes a post-level ID, a ticket-level ID, and variables indicating what platform the post appeared on, whether the post is labeled, whether the poster is verified, post language, whether the post is a standalone post, a reply, a quote tweet, a retweet, or a Facebook re-share, whether the post includes an external URL, whether the post includes an image or video, and whether embedded content was labeled or removed.

B. Categories: In-scope narratives

The Election Integrity Partnership flagged the following types of narratives:

- Procedural interference: Misleading content related to actual election procedures, e.g. the mechanics of voting (time, place, and manner of voting).
- Participation interference: Content designed to deter participation in the election process, e.g. threats to personal safety.
- Fraud: Content that encouraged people to misrepresent themselves to affect the electoral process or illegally cast or destroy ballots.

- Delegitimization: Content that aimed to delegitimize election results on the basis of false or misleading claims.
- Premature claims of victory: Content that called the results of an election before they were officially called by an authoritative source.
- Calls to violent action: Specific calls that could lead to violence, specific claims of violence from the "other side" that are not true or use misleading evidence, or hints of violence if the poster does not get their way.

C. Facebook and Twitter's labels

Figures 1 and 2 show some of the labels the platforms applied ahead of the 2020 election.

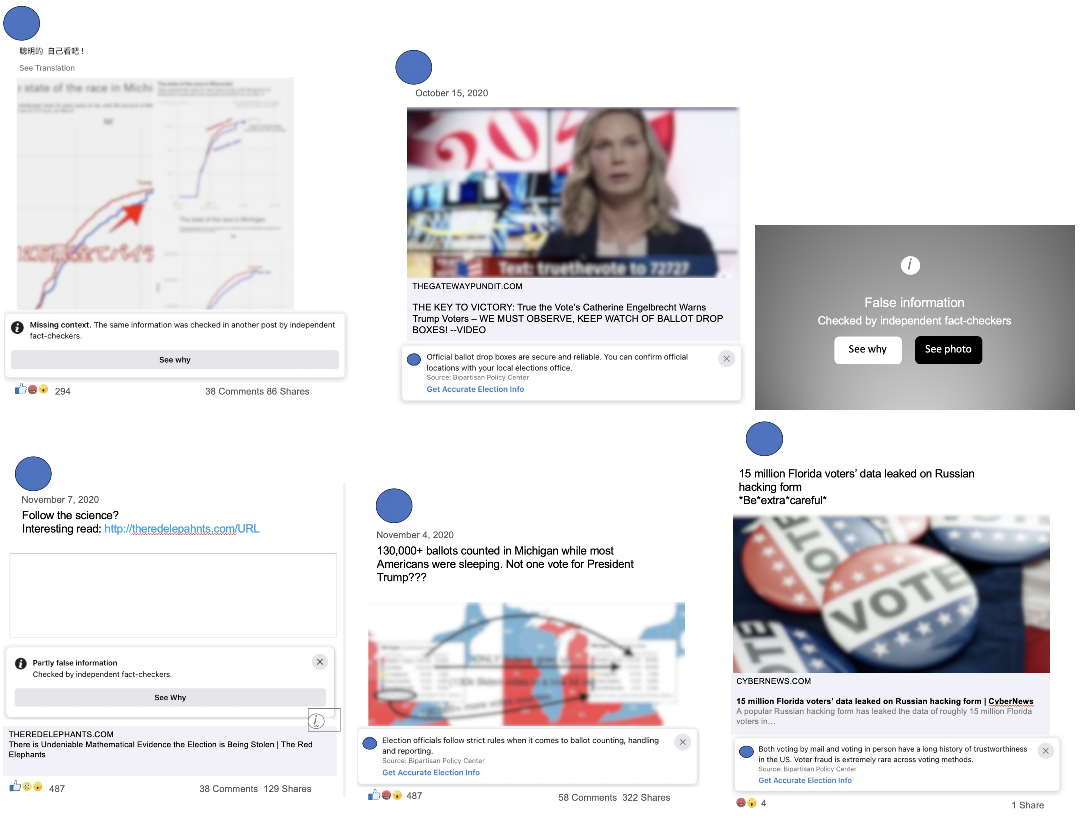

Figure 1: Labels Facebook applied ahead of the 2020 election. Note: figures have been recreated.

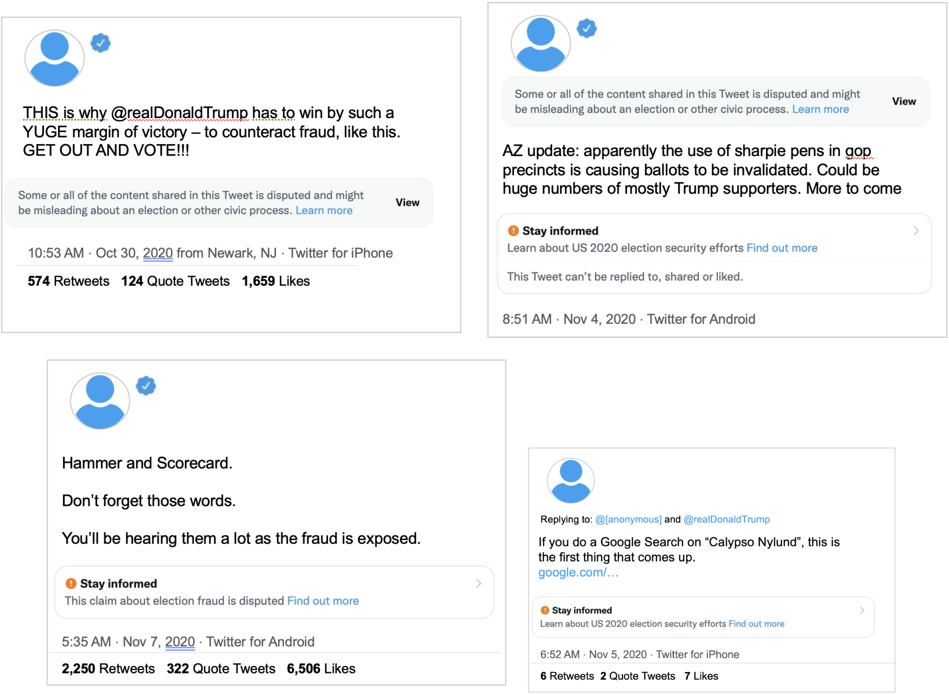

Figure 2: Labels Twitter applied ahead of the 2020 election. Note: figures have been recreated.

D. Script for assessing platform action

While we manually evaluated the status of all posts in the dataset for this paper, we also wrote a script to automate the process of loading and assessing posts in our dataset. We report on this process here as it was highly accurate and may be useful for future researchers. We ran this script first on December 7, 2020 to provide real-time findings for the Partnership final report. However, at the time we were not able to manually double check the script. For this paper we rely on the dataset created as a result of our manual inspection on June 4, 2021. The disadvantage of this decision is that our dataset may not reflect enforcement measures that were taken in the run-up to the election or soon afterwards; it may instead reflect enforcement that took place weeks later. However, we prefer to use the more accurate dataset, and believe it is unlikely that platforms labeled or removed much content weeks after the election.

The script scraped every single post in the dataset and checked whether:

1. the content was on a relevant platform (Facebook, TikTok, Instagram, Twitter, or YouTube),

2. the content was labeled, or

3. the content was removed.

The script worked by launching a “headless” instance of Chrome (version 91) that visited each post and relayed information about the page back to the script, which was then used to determine how the content was actioned. The script processed what a U.S.-based visitor would encounter when visiting the posts. To bypass “login walls” on Facebook and Instagram, our script authenticated with those platforms using a real user account.

While we experimented with extracting engagement data (number of likes, shares, views, etc.) from each post to supplement our analysis, we found that the anti-scraping measures employed by the platforms made this difficult to do reliably. Additionally, since the script examines each post in isolation, it has no way of detecting less overt moderation actions (such as downranking).

The script used textual signals (e.g., the presence of particular text on a page, such as ‘This Content Isn’t Available Right Now’), visual signals (e.g., the presence of a particular warning icon used in content labels), and semantic signals (e.g., the underlying structure of the web page) to determine the correct coding. The script also took a screenshot of every post in the dataset from a relevant platform so that its codings could be easily verified. Our manual review process largely relied on these screenshots.

The script performed reasonably well; when we tested it on the 4,681 posts in June 2021 and compared its results to our manual results, it made mistakes on only 163 URLs — mostly due to multiple tweets appearing on the same page (e.g., in a thread). Note that these numbers reference the full post dataset, not the analysis dataset used in this paper.

E. Platform policies

Platform policies are spread across community standards, terms of service, and policy change blog posts that may not be incorporated into its Community Standards (Miller, 2021), which makes it difficult to pinpoint what they prohibit. In this section we describe relevant platform policies in more detail. See Appendix F of Election Integrity Partnership (2021) for a detailed description of what policies changed across platforms.

Facebook’s Community Standards discuss election-related content under instructions “do not post” and when discussing groups that are not allowed on Facebook (Facebook, N.d.). The platform tells users not to post misleading content about voting, calls for actions that would interfere in an individual’s ability to participate in any aspect of an election, calls for violence related to any aspect of an election, calls to bring weapons to any place related to election (and, sometimes, to “certain locations where there are temporarily signals of a heightened risk of violence or offline harm. [...] for example, when there is a known protest and counter-protest planned or violence broke out at a protest in the same city within the last 7 days”), threats against election officials, attacks on institutions or practices that may lead to harm particularly during elections, and expressions of support for intimidating voters. Facebook says it does not allow groups that repeatedly threaten to “violently disrupt

an election process.” In September 2020 a Facebook blog post (much of which has not been incorporated into its Community Standards) announced that it would remove posts that use COVID-19 to discourage voting, and label posts that seek to delegitimize aspects of the election (Meta, 2020). The blog post was unique in describing enforcement actions for violative content; the Community Standards do not do this explicitly.

Twitter’s election policies fall under its Civic Integrity Policy (Twitter, 2021). It summarizes its policy as prohibiting posts that “may suppress participation or mislead people about when, where, or how to participate in a civic process. In addition, we may label and reduce the visibility of Tweets containing false or misleading information about civic processes in order to provide additional context.” The policy covers four categories of content: Misleading information about how to participate, suppression and intimidation, misleading information about outcomes, and false or misleading affiliations. Twitter also prohibits accounts with a false or misleading affiliation. Twitter’s violent threats policy forbids “[threatening] violence against an individual or a group of people” and “the glorification of violence” (Twitter, 2019) and a blog post specific to the election says that it will remove tweets that call for violence (Gadde, 2020).

F. Multilingual analysis

While the vast majority of the user-generated content that we analyzed was in English, our analysis does include some content not in English (as shown below in Figure 1. We used Google Translate to translate non-English content for our analysis.

G. Post language and label

	Facebook		Twitter	
**Language**	**Label**	**No label**	**Label**	**No label**
Arabic	2	0	0	0
Chinese	2	19	0	7
English	147	226	42	545
Finnish	1	0	0	0
French	4	0	0	1
Hebrew	1	0	0	0
Hungarian	1	18	0	1
Japanese	0	0	1	0
Portuguese	1	0	0	2
Romanian	1	0	0	0
Spanish	2	3	2	2
Vietnamese	0	3	0	0
No language	1	0	0	0

Table 1: Number of posts across platforms by language, and whether the post was labeled.

References

Election Integrity Partnership. 2021. *The long fuse: Misinformation and the 2020 election*. v1.3.0 ed. Stanford Digital Repository: Election Integrity Partnership.
URL: *https://purl.stanford.edu/tr171zs0069*

Facebook. N.d. "Facebook community standards." https://transparency.fb.com/policies/community-standards/. (Accessed on 05/23/2022).

Gadde, Vijaya, Kayvon Beykpour. 2020. "Additional steps we're taking ahead of the 2020 US election.". (Accessed on 05/23/2022).
URL: *https://blog.twitter.com/en_us/topics/company/2020/2020-election-changes*

Meta. 2020. "New steps to protect the US elections.". (Accessed on 05/23/2022).
URL: *https://about.fb.com/news/2020/09/additional-steps-to-protect-the-us-elections/*

Miller, Carly. 2021. "Facebook, it's time to put the rules in one place.". (Accessed on 05/23/2022).
URL: *https://www.lawfareblog.com/facebook-its-time-put-rules-one-place*

Twitter. 2019. "Violent threats policy." https://help.twitter.com/en/rules-and-policies/violent-threats-glorification. (Accessed on 05/23/2022).

Twitter. 2021. "Civic integrity policy." https://help.twitter.com/en/rules-and-policies/election-integrity-policy. (Accessed on 05/23/2022).
